# Re-evaluating evidence for giant genomes in amoebae

**DOI:** 10.1590/1678-4685-GMB-2024-0092

**Published:** 2024-12-20

**Authors:** Daniel Barzilay, João P. B. Alcino, Giulia M. Ribeiro, Alfredo L. P. Sousa, Daniel J. G. Lahr

**Affiliations:** 1 Universidade de São Paulo, Instituto de Biociências, Departamento de Zoologia, São Paulo, SP, Brazil.; 2 Lund University, Department of Bioinformatics, Sölvegatan, Lund, Sweden.

**Keywords:** Genome evolution, next-generation sequencing, biodiversity, microbial diversity

## Abstract

Here we reassess available evidence for the long-held misconception of amoebae possessing exceptionally large genomes. Traditionally, estimates relied on inaccurate methods like DNA weight measurements, leading to inflated sizes. These methods failed to account for contaminating DNA from prey, endosymbionts, and intrinsic genomic features like ribosomal operon amplification. Modern sequencing techniques unveil a different picture. Fully sequenced amoebozoa genomes range from 14.4 to 52.37 mega basepairs, well within the typical single-celled eukaryote expectation. While the whole genome of the historically relevant *Amoeba proteus* has not yet been fully sequenced, we provide here a statistical analysis using protein-coding genes from transcriptomic data, suggesting that the genome size is consistent with this range, far smaller than previously claimed. The misconception likely originated in the early 21^st^ century and perpetuated through popular science materials. We conclude that there is no longer reason to reaffirm that amoeba genomes are giant.

## Amoebae are a paraphyletic biological group recognized by the ability to produce pseudopods

**Figure 1:**
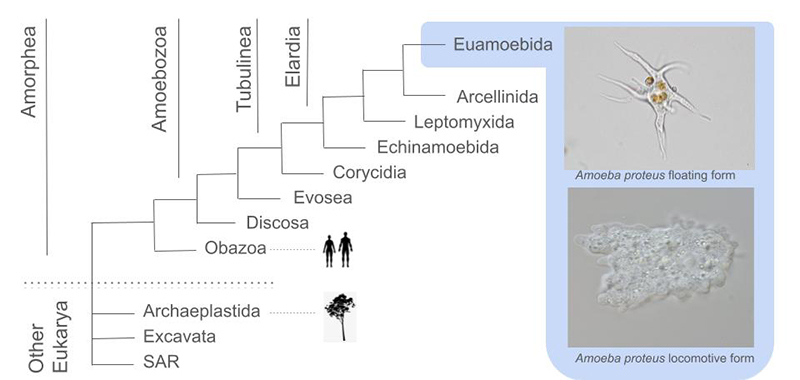
A simplified phylogenetic tree of eukaryotes, highlighting the major amoebozoan lineages, with emphasis on *Amoeba proteus*. The general tree topology is based on Burki, 2020 and is unrooted, because the root of eukaryotes is still unknown. The Amorphea part of the tree, and detailed Amoebozan affinities are extracted from Kang *et al.*, 2017. The text-book exemplary amoeboid organism, *Amoeba proteus*, is represented in its two most commonly shapes found in the environment, a floating form and a locomotive form. All of the animals are inside Obazoa, along with Breviata, Apusomonada, Fungi and other single-celled lineages. The Plants are in Archaeplastida, along with the green and red algae, among others.

An amoeba is a unicellular organism with the ability to dynamically alter its shape and movement. These plastic movements are executed by pseudopods, a temporary extension of the cytoplasm promoted mostly by an actin-myosin network ( Singleton and Sainsbury, 2007; Barger *et al.*, 2020). Since the original observation and record in the 18th century by August Johann Rösel von Rosenhof (described as “ *the Little Proteus*”), ( Lorch, 1973) thousands of other free-living and a handful of parasitic amoebae have been described by scientists ( Adl *et al.*, 2019). Until the end of the 20^th^ century, all amoeboid organisms were classified as Sarcodina, other similar taxon names include Rhizopoda and Lobosa, depending on the system ( Bovee, 1985). The inclusion of diverse organisms within the same taxonomic group indicates that the consensual interpretation was that these organisms would have a single evolutionary origin in the history of life. However, contemporary phylogenetic studies based on molecular genetics have dismantled Sarcodina ( Figure 1). In fact, most rhizopoda (or sarcodines, or loboseans) are currently placed in two major, vastly diverging, eukaryotic lineages: the Amoebozoa and the Rhizaria, while many others are scattered across the tree of eukaryotes such as in Stramenopila, Opisthokonta and more ( Burki *et al.*, 2020). These groups are distantly related to each other in the tree of life, as such, the current interpretation is that the amoeboid habit has independently and convergently appeared many times in the history of evolution ( Adl *et al.*, 2019).

Some amoebae are familiar to the general biologist audience, featuring as exemplary single-celled organisms in biology text-books: *Amoeba proteus,* a typical example of a free-living amoeba; *Entamoeba histolytica* a typical example of a parasite, and; *Acanthamoeba* spp. representing the neglected opportunistic pathogens ( Hickman *et al.*, 2008). All of these examples are currently classified as Amoebozoa, along with the curious social slime-mold *Dictyostellium discoideum*, a model organism ( Martín‐González *et al.*, 2020). Biology students will often come into contact with multiple other examples of amoebae, such as the Foraminifera, currently classified in the Rhizaria, an amoeboid lineage that produces intricate shells with geo-prospecting importance ( Weiner and Dove, 2003). Aside from these classical examples, the diversity of amoebae is much wider than generally thought. For instance, Amoebozoa alone comprises more than 20 major lineages, and the species richness within these lineages may reach tens of thousands of species ( Kang *et al.*, 2017). However, most biologists will never come into contact with the more practical aspects of amoebae research: the vast majority of lineages are simply non-culturable; sampling and identifying species are challenges that require years of laboratory training to be overcome, and; methods for cellular and molecular study are not easily transferred to these non-model, heterogenous organisms. These restrictions impose several challenges to advancing scientific knowledge in the group, though single-cell transcriptomic sequencing has brought new insights ( Lahr *et al.*, 2019). 

## The myth of giant amoeba genomes was introduced by a single paper and propagated, despite many specialist critiques through decades

Measures of genome size have varied historically. Most sources agree that a useful standard measure is the total amount of DNA contained within the haploid complement of a given genome, canonically termed the C-value ( Greilhuber *et al.*, 2005). Contemporarily, C-value is estimated directly from whole genome sequencing, but not too long ago, methods included DNA extraction, purification and weighing, to estimate total size, in this equation 1 pg roughly translates to 978 million basepairs ( Greilhuber *et al.*, 2005). This method was cheap and easy, but highly inaccurate because not only is it an indirect estimation of DNA content, it is also very hard in non-model organisms to separate *ex ante* foreign contaminants and guarantee the cell is haploid (or diploid!), leading to overestimation of DNA size ( Greilhuber *et al.*, 2005). That is why direct methods like DNA sequencing and *ex post* filtration of foreign DNA yields a more accurate estimate. However, both cost and quantity of pure material are a limitation for these methods. 

There are two kinds of issues when estimating amoeba genome sizes. The first kind relates to intrinsic biological characteristics of the organisms. The second kind stems from methodological problems. In this section, we will summarize the main empirical works and their approaches, while pointing out methodological concerns. Further, we will address biological aspects that are common to all approaches.

## The original estimate of a giant amoeba genome size was experimentally inaccurate, and many researchers have pointed that aspect multiple times

Carl T. Fritz provided some of the first measurements of amoeboid genomes using the weighted fraction measurement method ( Fritz, 1968), for organisms identified by the authors as *Chaos chaos, Amoeba dubia* and *Amoeba proteus* species. The method indirectly estimates genome size by filtering and fractionating nuclei in a suspension containing approximately 5 × 10⁶ cells, followed by weighing. DNA and RNA weights are then used as proxies for estimating genome size. The author concluded that each individual *C. chaos* had a total DNA weight of 1.4 ng, and *A. dubia* with 0.7 ng and *A. proteus* 0.3 ng (do note that the author uses an outdated notation of mμg, which we assume translates currently to ng). These values indicate that, if a *C. chaos* had a single nucleus and that nucleus was diploid, we would assume 0.7 ng in the haploid complement of its genome, which translates to about 685 billion basepairs (Gigabases). Similarly, *A. dubia* would amount to 342 GB and *A. proteus* to 147 GB. Fritz did suggest that because *C. chaos* was multinucleated, the values should be corrected to reflect that, and offers the figure of a nuclear content that is 1/70 of that contained in *A. dubia*, which would translate to ~10 GB. 

An important context regarding amoeba names deserves mentioning: the taxonomic identification of amoeboid organisms is a centuries-old challenge. This is because cultures are unstable, and there are no reliable methods to preserve useful long-term name-bearing types, or vouchers ( Lahr *et al.*, 2012). The vast majority of cultures for experiments in the last century have been lost, and original authors have provided little to no evidence that would allow an objective identification of the organism they were working on. Current consensus is that Fritz’s original organism was most likely an exemplary in the Euamoebida lineage, but determining even a Genus is nearly impossible. Fritz does mention that his *Chaos chaos* was multinuclear, and that both *Amoeba* species were mononuclear, which indicates that the genera of the three are in agreement with current interpretation. 

Currently, Fritz’s experimental method is widely considered inaccurate and has been abandoned, at the same time, the specific interpretation given by the author was called into question by many others and reviewed in Byers ( 1986), who indicated that the problem was the large amount of foreign DNA especially since the prevailing cultivation method of *A. proteus* was to use the ciliate *Tetrahymena* as prey: ciliates have nuclear dualism, with a germline micronucleus and a somatic, highly polyploid macronucleus ( Tautvydas, 1971). Byers then produces an estimate using different strains of *Amoeba proteus* by isolating nuclei, ranging from 34 - 43 pg per nucleus (this is about 10 times less than Fritz’s original estimate). This estimate is in range with others, such as those offered by Afon’kin of 14 pg in A. proteus and 11 pg in C. chaos ( Afon’kin, 1989) *.*

Byers and others ( Spear and Prescott, 1980) tracked DNA synthesis through the life-cycle, and stated that “it is possible that part or all of the amoeba satellite DNA is rDNA”. Byers might have correctly predicted what we currently understand as an “extrachromosomal ribosomal operon”, which is present in many Amoebozoa lineages with a fully-sequenced genome available ( Glöckner and Noegel, 2012). This phenomenon arises when a dedicated, small chromosome encodes the full ribosomal operon, many times in two palindromic copies. This genomic piece is still inside the nucleus and typically undergoes many rounds of duplication, thus producing a large amount of repeated DNA and inflating estimates that are based on weight.

Further, amoebae are predatory organisms. With few exceptions ( *Entamoeba* and *Acanthamoeba*), cultures are non-axenic, also containing multiple eukaryotic and bacterial prey ( Pickup *et al.*, 2007). Even the best filtering techniques cannot isolate amoebae from other organisms in the culture, therefore the obtained DNA using this method will be that of the amoebae with additional DNA from a large number (perhaps even astronomical) of prey organisms, affecting genome size estimates. Additionally, large amoebae often harbor bacterial endosymbionts and undigested prey in their cytoplasm, further complicating measurements ( Greub and Raoult, 2004). Finally, many amoebae undergo ploidy cycles, which can significantly interfere with size estimation ( Parfrey *et al.*, 2008) unless some type of life-cycle phase correction has been executed ( Demin *et al.*, 2020).

In conclusion, the original estimates of amoeboid genome size that were based on DNA weight methods are overestimations most likely due to contaminant endosymbiont and prey DNA, but are also biased due to intrinsic genomic architecture mechanisms such as full-genome polyploidization, or copy number variation of a specific chromosome encoding the ribosomal operon.

## Chromosome numbers as a proxy for genome size have yielded contradictory conclusions in amoebae

Historically, chromosome number served as a secondary, independent method for estimating genome size in amoebae. This is a historical issue: perhaps earlier researchers reasoned that more chromosomes should mean a larger genome. Importantly, many of these reports were executed long before the concept of a C-value was established, as such, the possibility that many chromosomes could also mean high ploidy has not been taken into account in most works. Some reports suggested that *Amoeba proteus* could possess 500 or more chromosomes, originating in a seminal article by Werner Liesche (Liesche, 1938), using early methods of karyotyping which were microscopy-based and depend on large numbers of individuals to derive conclusions. However, amoeboid organisms are difficult to use in these conditions, since most cultures are asynchronous, as such the brief duration of the haploid phase limits the acquisition of real experimental data. The report by Werner Liesche has been challenged many times in history, on different grounds. Multiple researchers demonstrated that diverse amoeboid lineages are autopolyploid and go through rounds of full-genome replication ( Ord, 1968; Afon’kin, 1989; Makhlin, 1993; Demin *et al.*, 2019). More recently the phenomenon of chromatin extrusion (a drastic reduction of ploidy by discarding DNA into the cytoplasm) was demonstrated ( Goodkov *et al.*, 2019), as previously predicted by Parfrey *et al.* ( 2008).

To address these challenges and conflicting reports, Mariia Berdieva and colleagues employed new techniques to karyotype Amoeba proteus ( Berdieva *et al.*, 2019). This involved synchronizing culture phases, enlarging chromosomes with saline solutions to enhance visibility under optical instruments, and developing a novel technique to extract and spread chromosomes without damage by disrupting the nuclear envelope ( Demin *et al.*, 2017). Their findings revealed that *A. proteus* strain B possesses 27 pairs of chromosomes, each pair exhibiting homologous patterns of chromomere bands. This significantly contrasts with the previously speculated 500 or more chromosomes, aligning *A. proteus* more closely with other well studied Amoebozoa and providing valid experimental evidence that the genome is not of giant proportions: while chromosomes may vary in size, with 27 pairs and an original estimate of a 147 GB c-value, a single *A. proteus* chromosome would be about 5.44 GB, which is close to twice the size of the entire human genome.

## Exaggerated and disputed claims for giant amoeba genome sizes were adopted and propagated in non-specialist circles

Perhaps the most insidious aspect of this historical sequence of inaccurate estimations of amoeboid genome sizes, is their introduction into educational material and scientific popularization books. Unfortunately, this is a much harder trail to reconstruct. These types of sources are not as well indexed as peer-reviewed scientific articles. The most relevant of them will be undergraduate or high-school level text-books printed in a variety of native languages, and access to them is quite complicated, even in the era of hyper communication. We can find examples over recent decades, such as in scientific popularization magazines in Spain ( *Magazine Metòde* - Latorre and Silva, 2013) and Brazil ( *Revista FAPESP* - Fioravanti and Pivetta, 2001), local newspapers ( *Folha de São Paulo* - Bonalume Neto, 2003), but determining exactly how widespread this idea is popularly will be a difficult exercise. The idea certainly has penetration into the scientific mindset, a curious recent example can be pointed out from the most recently published amoeboid genome, which intriguingly is the smallest tubulinean genome to date, is juxtaposed by the authors as a conundrum:

*“Traditionally recognized as a clade possessing large genome sized members (* *Fritz, 1968* *), our study reveals the smallest free-living amoeba genome within the Tubulinea clade”.* ( Tekle and Tefera, 2024)

We estimate that this myth originates in the early 21st century. The first traceable popular reference is from a science dissemination book originally printed in 2005 ( Gregory, 2005), which presents an image that became quite ubiquitous in text-books and websites (Figure 1.1 in the book), eventually making it into Brenner’s Encyclopedia of Genetics ( Sessions, 2013, pp. 301-305). Gregory correctly indicates the genome size in weight, as proposed by Fritz, and explicitly points out many of the same biases raised here for the likely overestimation of amoeboid genome sizes. Subsequent authors typically raise the same issues, but at some point, the estimate of genome size is transformed into a C-value. The idea is then propagated as a fun interesting fact, often mixed with solid genome research data for other eukaryotic species like humans, rats, plants ( Johnson, 2007). Consequently, this myth solidified through generations of scientists in their formative years. References to the idea seem to appear more strongly during the years following the finalization of the human genome sequencing. 

## Amoeba genomes have legitimate biological processes for increase in genome complexity, not necessarily size

A high number of foreign gene acquisitions have been putatively identified in Amoebozoa genomes. Amebae are grazers of environmental bacteria and also have obligatory, transient and permanent associations with bacteria that participate in their metabolism ( Ribeiro and Lahr, 2022). That relationship may lead to a high number of lateral gene transfers, reaching up to 15% of the genome, which would still not account for an increase of orders of magnitude in genome size ( Tekle *et al.*, 2022).

Another source of genomic alteration via lateral gene transfer in amoebae are their relations with viruses, notably giant viruses. Since the discovery of mimivirus, numerous giant viruses associated with free-living amoebae have been described. The genome of giant viruses can be more than 2.5 megabases, and virus particles can exceed the size of many bacteria. The main hosts of giant viruses (e.g., tupanvirus, faustovirus and kaumoebavirus, ( Dorine *et al.*, 2015; Bajrai *et al.*, 2016; Silva *et al.*, 2019) are species in the genus *Acanthamoeba* (a free-living predator that can act as an opportunistic human pathogen), and the free-living *Vermamoeba vermiformis* ( Visvesvara *et al.*, 2007; Delafont *et al.*, 2013). The current paradigm indicates that these amoebae are infected with viral particles during phagocytosis ( Andrade *et al.*, 2017). Conversely, viral genomes also bear evidence of incorporation of amoebae genes into their genome ( Schulz *et al.*, 2017).

## Well characterized amoeba genomes are not giant

**Figure 2 -:**
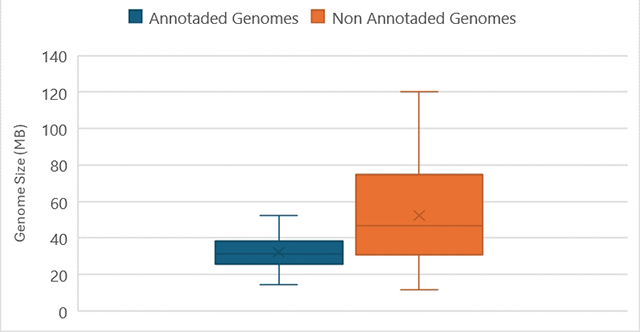
Genome sizes of the 65 Amoebozoan genomes deposited in GenBank, as of April 2024. The blue boxplot represents the 15 annotated reference genomes, and the orange boxplot represents the additional 50 deposited genomes for which there is no annotation available.

A number of species of amoebozoans have been sequenced in the past 2 decades, but genomic analyses have been mostly limited to model organisms or medically important lineages. Consequently, the vast diversity of Amoebozoa genomes still remain largely unexplored, and an effort into sequencing diverse amoebae would be more than welcome ( Table S1, Figure 2) - for instance, the subgroup Tubulinea is very undersampled, with only three non-reference representatives ( *Vermamoeba vermiformis, Trichosphaerium sp.* and *Echinamoeba sylvestris*). The first few genomes were generated using traditional Sanger-sequencing methods, as well as high-throughput sequencing platforms such as 454 and Ion torrent. These methods present challenges for complete assembly of the genome via bioinformatics, mainly due to repeating elements, especially without a reference genome. With the advances in the last decade sequencing using Illumina and Nanopore platforms, among others, yielded genomes with a higher accuracy and coverage allowing for better annotations of the data, showing the amount of proteins coding genes and replication machinery genes, with a higher degree of certainty ( Table S2, Figure 2).

## Estimating genome-sizes based on transcriptomes indicates that the *Amoeba proteus* genome is not giant

**Figure 3 -:**
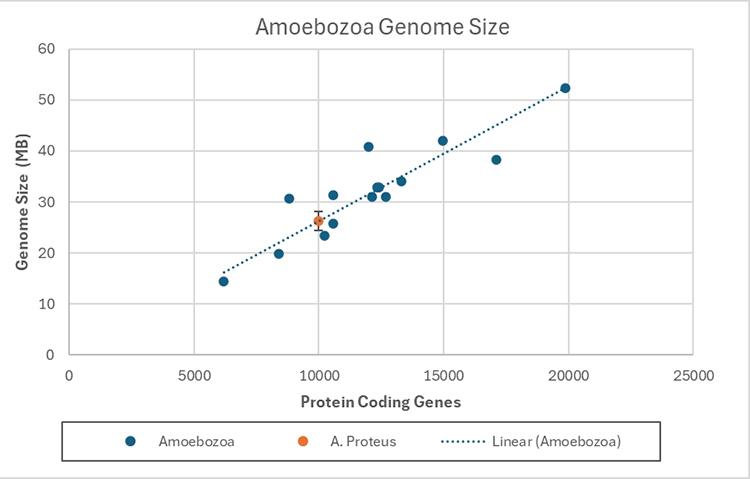
Linear regression of Number of Protein-coding genes against Genome size for annotated reference Amoebozoa genomes, and projection of Genome size for *Amoeba proteus*. All genomes used for this regression are representatives of Evosea, with the exception of *Acanthamoeba* in the Discosea. Regression formula: Genome size (MB) = Total number of Protein coding genes X 0,002636 (std error 1.82714).

After much controversy over the years, the vast amounts of sequenced genome data enabled the characterization of a strong linear relationship between number of protein coding genes and genome size for eukaryotic organisms ( Hou and Lin, 2009; Titus-McQuillan *et al.*, 2023). Using this observation, we selected from NCBI all amoebozoa reference genome sequences (n=15). The recently published smallest tubulinean genome for *Echinamoeba silvestris* (Accession number PRJNA1047129) has not been included, because it is not yet publicly available at NCBI as of the writing of this manuscript. We then performed a least squares regression model of Genome size in Mega Bases (MB) against the total number of protein coding genes (we used the official NCBI estimate of numbers of genes available from each accession). This resulted in a very strong linear relationship ( Figure 3, R² = 98.55%). 

With a model in hand, we estimated the number of genes in the *Amoeba proteus* genome from a previously published transcriptomic dataset. The dataset was published by our research group in 2017 ( Kang *et al.*, 2017), and we summarize briefly here the methodological steps taken to reduce the amount of contaminating data. Firstly, single cells were isolated from nature and subjected to taxonomic identification, including photo documentation (vouchering) of the exact individual to be sequenced. These organisms were then individually cleaned through serial dilution and stored in sterile media for at least 12 hours so that all (or most) prey material was digested. The cells were then, one by one, subjected to lysis and a protocol to enrich poly-adenylated mRNA, and then proceeded to sequencing ( Picelli *et al.*, 2014). The nucleic acid was fully sequenced using an Illumina platform. The sequence reads were then subjected to a bioinformatics pipeline that eliminated environmental carry-over (bacterial genetic material), assembled the transcripts and then analyzed data in an eukaryotic phylogenetic context ( Tice *et al.*, 2021). The final dataset was then mostly free of non-eukaryotic contamination, and from this dataset a total number of protein-coding genes can be derived.

Using this data in the above formula we estimated the genome size of *A. proteus* should be between 24.5 MB and 28.1 MB. This number is four orders of magnitude smaller than 296 GB and in line with other amoebozoa species. It is noteworthy that tubulineans are poorly represented genomically (only three available draft genomes), and have been completely excluded from our linear regression analysis, because there are no reference genomes. However, each individual (draft) tubulinean genome does not present any indication of an abnormally large genome size, in fact, the recently published *Echinamoeba sylvestris* (a tubulinean) genome is among the smallest in Amoebozoa.

## Conclusion

We have demonstrated here that there is no factual reason to think amoebozoa genomes should be exceptionally large. Firstly, all amoebozoans with a fully sequenced genome have a C-value between 14.40 and 52.37. Further, taking into account the natural history of ameba species as predators of bacteria and hosts of a wide variety of endosymbionts (including both bacteria and giant viruses), indicates that direct measures of DNA weight from amoebal cells will inevitably overestimate the amoeba’s genome size. Additionally, most amoebae are autopoliploid, and all amoebae that have a fully sequenced genome encode their ribosomal operon in a single smaller and circular chromosome that is copied many times. These observations indicate that measures of genome size based on isolated nuclei are also compromised. Finally, we present a statistical projection based on protein-coding gene numbers extracted from transcriptomic studies. Our projections indicate that the genome size of the classical *Amoeba proteus*, that has been historically implicated in claims of giant genome size, should fall within the normal range of microbial eukaryotes. It is our opinion that we do not have to wait for the sequencing of diverse dozens of free-living amoeba genomes to be published before we can put the idea of giant amoeba genomes to rest.

## Supplementary material

The following online material is available for this article:

Table S1 Non Annotated Genome from NCBI. This table lists every Amoebozoa genome deposited at NCBI.

Table S2 Annotated Reference Genome from NCBI. This table lists every Amoebozoa genome available in NCBI, with a reference status.
