# Supplementary material for: Re-evaluating evidence for giant genomes in amoebae
Source: Genet Mol Biol. 2024 Dec 20;47(Suppl 1):e20240092. doi: 10.1590/1678-4685-GMB-2024-0092 (PMC11773323; doi:10.1590/1678-4685-GMB-2024-0092)
Supplement: Table S1 [file 1415-4757-GMB-47-s1-e20240092-s1.pdf]

Genetics and Molecular Biology
https://doi.org/10.1590/1678-4685-GMB-2024-0092

Supplementary Material to "Ending the myth of giant amoeba genomes: Re-evaluating evidence for giant genomes in amoebae"

Table S1 - Non Annotated Genome from NCBI. This table lists every Amoebozoa genome deposited at NCBI. We provide accession number, genome size, type of assembly and date for each entry.

Non Annotated Genome NCBI				
**Assembly Name**	**Organism Name**	**Size (MB)**	**Assembly Level**	**Assembly Date**
ASM2069642v1	Vermamoeba vermiformis	39,46	Scaffold	01/11/2021
ASM3183524v1	Balamuthia mandrillaris	65,2	Contig	25/09/2023
mastiga_genome_v5.1	Mastigamoeba balamuthi	57,27	Scaffold	14/11/2019
ALPT14_1.0	Acanthamoeba lenticulata	75,57	Contig	08/06/2017
ASM291457v1	Entamoeba moshkovskii	25,25	Scaffold	02/02/2018
ASM2422052v1	Cochliopodium minus	50,55	Scaffold	12/07/2022
ASM1337569v1	Dictyostelium brefeldianum	37,13	Scaffold	29/06/2020
ASM1525263v1	Dictyostelium capitatum	46,73	Scaffold	06/11/2020
PRJEB36491	Acanthamoeba triangularis	66,43	Contig	14/02/2021
ASM27746v1	Dictyostelium intermedium	30,53	Scaffold	10/07/2012
Dcit_1.0	Dictyostelium citrinum	26,25	Scaffold	01/11/2012
ASM1525267v1	Dictyostelium gargantuum	43,16	Scaffold	06/11/2020
ASM215122v1	Paramoeba pemaquidensis	34,39	Scaffold	19/05/2017
DUN_Pmulti_V1.0	Heterostelium multicystogenum	30,33	Contig	19/10/2018
ASM2794324v1	Acanthamoeba sp. SK_2022b	49,33	Contig	23/01/2023
ASM2794329v1	Acanthamoeba sp. SK_2022a	45,79	Contig	23/01/2023
ASM208155v1	Protostelium mycophagum	39,72	Scaffold	06/04/2017
Acanthamoeba royreba	Acanthamoeba royreba	79,54	Scaffold	07/01/2015
ASM2334592v1	Vannella sp.	14,24	Contig	12/05/2022
Acanthamoeba culbertsoni genome assembly	Acanthamoeba culbertsoni	55,54	Scaffold	06/01/2015
Acanthamoeba healyi	Acanthamoeba healyi	75,32	Scaffold	07/01/2015
Acanthamoeba divionensis	Acanthamoeba divionensis	84,77	Scaffold	07/01/2015
Acanthamoeba astronyxis	Acanthamoeba astronyxis	83,43	Scaffold	07/01/2015

Non Annotated Genome NCBI				
**Assembly Name**	**Organism Name**	**Size (MB)**	**Assembly Level**	**Assembly Date**
ASM156762v1	Acanthamoeba polyphaga	49,35	Contig	24/02/2016
Acanthamoeba quina	Acanthamoeba quina	83,59	Scaffold	07/01/2015
Acanthamoeba mauritaniensis	Acanthamoeba mauritaniensis	106,84	Scaffold	07/01/2015
KYO_Dcav_2	Speleostelium caveatum	30,95	Scaffold	19/10/2018
Acanthamoeba rhysodes	Acanthamoeba rhysodes	75,82	Scaffold	07/01/2015
Acanthamoeba lugdunensis	Acanthamoeba lugdunensis	99,42	Scaffold	07/01/2015
ASM202528v1	Acanthamoeba comandoni	86,21	Contig	13/03/2017
Acanthamoeba pearcei	Acanthamoeba pearcei	115,61	Scaffold	07/01/2015
ASM2794497v1	Acanthamoeba sp. SK_2022c	15,7	Contig	23/01/2023
BN839	Mastigamoeba balamuthi	48,59	Contig	09/10/2013
Assembly 1	Entamoeba histolytica HM-1:IMSS	25,2	Scaffold	15/10/2021
ASM1846681v1	Entamoeba histolytica DS4-868	19,76	Contig	24/05/2021
ASM2028350v1	Entamoeba histolytica MS96-3382	19,02	Contig	05/10/2021
ASM1905953v1	Entamoeba histolytica KU48	16,68	Contig	29/06/2021
ASM2028353v1	Entamoeba histolytica KU50	11,89	Contig	05/10/2021
ASM2102059v1	Acanthamoeba castellanii	46,15	Contig	01/12/2021
ASM2102060v1	Acanthamoeba castellanii	43,83	Contig	01/12/2021
Acas_2.0	Acanthamoeba castellanii	46,71	Scaffold	22/03/2011
PRJEB38202	Acanthamoeba castellanii	69,07	Contig	20/07/2020
ASM118514v1	Balamuthia mandrillaris	67,62	Scaffold	22/07/2015
ASM126247v1	Balamuthia mandrillaris	44,27	Scaffold	05/08/2015
ASM27748v1	Dictyostelium firmibasis	30,56	Scaffold	10/07/2012
Acanthamoeba lenticulata	Acanthamoeba lenticulata	66,03	Scaffold	07/01/2015
KYO_Dpur_P1Ba_4	Dictyostelium purpureum	32,32	Scaffold	12/10/2018
ASM210525v1	Acanthamoeba lenticulata	74,7	Contig	21/04/2017
Acanthamoeba polyphaga	Acanthamoeba polyphaga	120,42	Scaffold	08/01/2015
ASM2334648v1	Vannella sp.	11,66	Contig	12/05/2022
