# Supplementary material for: Re-evaluating evidence for giant genomes in amoebae
Source: Genet Mol Biol. 2024 Dec 20;47(Suppl 1):e20240092. doi: 10.1590/1678-4685-GMB-2024-0092 (PMC11773323; doi:10.1590/1678-4685-GMB-2024-0092)
Supplement: Table S2 [file 1415-4757-GMB-47-s1-e20240092-s2.pdf]

Genetics and Molecular Biology
https://doi.org/10.1590/1678-4685-GMB-2024-0092

Supplementary Material to "Ending the myth of giant amoeba genomes: Re-evaluating evidence for giant genomes in amoebae"

Table S2 - Annotated Reference Genome from NCBI. This table lists every Amoebozoa genome available in NCBI, with a reference status. Each line is a hyperlink to the accession at NCBI, and we provide genome size, number of genes and number of protein-coding genes estimated for each genome.

Annotated Reference genome NCBI					
**Assembly Name**	**Scientific name**	**Size (Mb)**	**Genes**	**Protein-coding**	**Phylum**
dicty_2.7	Dictyostelium discoideum AX4	34.15	13961	13289	Evosea
ASM3616959v1	Dictyostelium firmibasis	31.40	11044	10564	Evosea
JCVI-ESG2-1.0	Entamoeba histolytica HM-1:IMSS	20.84	8327	8151	Evosea
Acastellanii.strNEFF v1	Acanthamoeba castellanii str. Neff	42.02	15650	14968	Discosea
EIA2 v2	Entamoeba invadens IP1	40.88	12007	11997	Evosea
DFas_2.0	Cavenderia fasciculata	31.02	12333	12135	Evosea
JCVI_EDISG_1.0	Entamoeba dispar SAW760	30.63	8814	8811	Evosea
v1.0	Dictyostelium purpureum	32.97	12399	12395	Evosea
ENU1 v1	Entamoeba nuttalli P19	14.40	6193	6187	Evosea
PolPal_Dec2009	Heterostelium album PN500	32.97	12590	12336	Evosea
Asub_2.0	Acytostelium subglobosum LB1	31.00	12682	12682	Evosea
